# Dissolution of Volcanic Ash in Alkaline Environment for Cold Consolidation of Inorganic Binders

**DOI:** 10.3390/ma17205068

**Published:** 2024-10-17

**Authors:** Giovanni Dal Poggetto, Philippe Douwe, Antonio Stroscio, Elie Kamseu, Isabella Lancellotti, Antoine Elimbi, Cristina Leonelli

**Affiliations:** 1 Department of Engineering “Enzo Ferrari”, University of Modena and Reggio Emilia, Via P. Vivarelli 10, 41125 Modena, Italy; giovanni.dalpoggetto@unimore.it (G.D.P.); antonio.stroscio@unimore.it (A.S.); elie.kamseu@unimore.it (E.K.); isabella.lancellotti@unimore.it (I.L.); 2 Department of Civil, Construction, and Environmental Engineering, North Carolina State University, Raleigh, NC 27695-7908, USA; 3 Department of Mathematics-Physics-Chemistry, University of Pala, Pala P.O. Box 20, Chad; philpsdouwe@yahoo.fr; 4 Department of Inorganic Chemistry, University of Yaoundé I, Yaoundé P.O. Box 334, Cameroon; elimbiantoine@gmail.com; 5 Department of Biological, Geological and Environmental Sciences, University of Catania, Corso Italia 57, 95129 Catania, Italy; 6 MIPROMALO—Local Material Promotion Authority, Yaoundé P.O. Box 2396, Cameroon

**Keywords:** dissolution kinetics, alkaline solution, volcanic ash, mechanical performance, room-temperature consolidation

## Abstract

A systematic study on the dissolution in concentrated alkali of two volcanic ashes from Cameroon, denoted as DAR and VN, is presented here. One volcanic ash, DAR, was 2 wt% richer in Fe and Ca and 4 wt% lower in Si than the other, designated as VN. Such natural raw materials are complex mixtures of aluminosilicate minerals (kaersutite, plagioclase, magnetite, diopside, thenardite, forsterite, hematite, and goethite) with a good proportion of amorphous phase (52 and 74 wt% for DAR and VN, respectively), which is more reactive than the crystalline phase in alkaline environments. Dissolution in NaOH + sodium silicate solution is the first step in the geopolymerisation process, which, after hardening at room temperature, results in solid and resistant building blocks. According to XRD, the VN finer ash powders showed a higher reactivity of Al-bearing soluble amorphous phases, releasing Al cations in NaOH, as indicated by IPC-MS. In general, dissolution in a strong alkaline environment did not seem to be affected by the NaOH concentration, provided that it was kept higher than 8 M, or by the powder size, remaining below 75 µm, while it was affected by time. However, in the time range studied, 1–120 min, the maximum element release was reached at about 100 min, when an equilibrium was reached. The hardened alkali activated materials show a good reticulation, as indicated by the low weight loss in water (10 wt%) when a hardening temperature of 25 °C was assumed. The same advantage was found for of the room-temperature consolidated specimens’ mechanical performance in terms of resistance to compression (4–6 MPa). The study of the alkaline dissolution of volcanic ash is, therefore, an interesting way of predicting and optimising the reactivity of the phases of which it is composed, especially the amorphous ones.

## 1. Introduction

The action of alkali on aluminosilicates, such as clays, has been studied since 1927 [1]. At that time, it was scientifically proven that, unless the alkali is very dilute (pH = 9), clays begin to decompose and form silicates and aluminates when immersed in alkaline solutions. Specifically, clays are readily soluble in sodium hydroxide, insoluble in calcium hydroxide, and intermediate in barium and potassium hydroxide. Sodium hydroxide attacks kaolin-group minerals more strongly than it does montmorillonite, metabentonite, or illite [2]. Metakaolin, obtained via the thermal treatment of kaolin, was more soluble in alkaline media than kaolin; results on various samples suggest the existence of at least one alkali–kaolin compound and one alkali–metakaolin compound as early as 1946 [3]. From clays and calcined clays, these experiments were extended to other aluminosilicates, fly ash, low-quality bauxites, clays, kaolins, and nephelines [4]. The dependence of partially crystalline aluminosilicate minerals on NaOH concentration has been demonstrated, indicating that, in a complex solid, chemical decomposition of the aluminosilicate network is a problematic issue [5]. A special class of aluminosilicates is represented by pozzolan or volcanic ash (VA), a material derived from volcanic eruptions, which is readily available in natural environments, and its use is mainly limited to supplementary cementitious material [6,7]. Since 13 years ago, interesting studies have been undertaken on the utilisation of natural pozzolan, such as volcanic ashes in the fabrication of geopolymer cements [8,9]. In particular, it was noticed that, when VA has a high proportion of amorphous phase (40–45 wt%), it exhibits a remarkable reactivity in highly alkaline environments [10].

At this point, it is necessary to delve into the details of the dissolution/degradation process of aluminosilicate in alkaline solution.

Alkali activation consists of the three following successive rate-limiting processes that control the reaction progress:

1. Dissolution or decomposition of the amorphous fraction of the aluminosilicate powder,
2. Classical Fick diffusion through a surface layer, and
3. Diffusive transport through a more complex gel structure (interstitial gel) [11].

The last two steps often overlap, leading some authors to suggest the existence of only two processes, with the second being a combination of (2) and (3) [12]. The dissolution kinetics of the aluminosilicate precursor determine the reaction kinetics and strength development of geopolymers. Some other models have been proposed, such as that of Valentini [13], where the variations in the reaction pathways that occur when using alkaline activators of different compositions and concentrations can be associated with different macroscopic behaviours in terms of mechanical performance and durability. Recently, the dissolution stage can be observed indirectly [14,15,16] or directly, as in the case of rice hull ash, geothermal silica, fly ash, metakaolin, and granulated blast furnace slag in NaOH at 0.05–0.3 M [17], fly ash at 4, 8, 12, and 16 M [18], and biomass-derived fly ash at 10 M [19].

When the alkaline dissolution of aluminosilicate occurs in highly concentrated suspensions or pastes, then the consolidation of the gel structure of stage 3 can occur and a novel 3D aluminosilicate network is formed. The final product, an alkali-activated material (AAM) or a geopolymer, when the CaO is very low, is a chemically and mechanically robust solid that can find a large number of applications [20], mainly in the domain of the building materials industrial sector [21,22]. Such a process of the alkali activation of aluminosilicate materials can be listed as a process of the “cold sintering technique” [23], which is attracting the interest of researchers and industrialists who are looking for more sustainable building materials [7,24]. In particular, geopolymers have attracted more attention in recent decades due to the low CO_2_ emissions generated during their production, their interesting physical and mechanical properties, and their excellent durability [25,26,27]. The properties of geopolymers mainly depend on the type of aluminosilicate used for their production, with the most common being metakaolin and fly ash [28].

In areas where volcanic ash is available, not considering the problems of recent eruptions [29], the alkaline activation of this material has been proposed since 2009 [30]. In these first studies, the consolidated material was thermally treated at 400 °C to improve its mechanical strength, while later, it was proposed to cure the demoulded solid at 90 °C [31,32,33]. Other methods have been proposed to increase its mechanical stability, including the addition of reactive aluminosilicates, such as laterite [34] limestone [10,35], metakaolin [36,37], kaolin, pozzolan and burnt lime [38], and finally, biomass-derived silica-rich ash [39].

The present study aims to understand the dissolution mechanism of volcanic ash after the immersion of its powder in sodium hydroxide solutions and the extent of such dissolution, with the objective of optimising its consolidation/geolpolymerisation at room temperature. To this end, a systematic study was carried out to determine the amounts of chemical elements (Al, Si, K, Ca, Mg, and Fe) leached from two different volcanic ashes originating from the “Cameroon Volcanic Line” [40] as a function of ash powder size, sodium hydroxide molarity, and immersion time. In order to investigate the dissolution mechanism of the complex volcanic ash compositions, both XRF and XRD were performed on the VA obtained after immersion in sodium hydroxide solutions. In addition, certain properties of the cured binders were measured in terms of chemical and mechanical stability in order to identify the optimum activation conditions. These results could make it possible to select the most suitable volcanic scoria for the production of geopolymeric building materials with reliable properties.

## 2. Materials and Methods

### 2.1. Volcanic Ashes

The two volcanic ashes used in this study, denoted, respectively, as DAR (grey) and VN (brown), were collected during the rainy season (September 2022) along the “Cameroon Volcanic Line” on the flanks of Mount Daran (latitude N 7°22′45.214″ and longitude E 13°29′53.068″ and altitude 1148 m) and Mount Vinala (latitude N 7°12′20.071″ and longitude E 13°36′17.493″ and altitude 1185 m) in the Adamawa Region of Cameroon [41]. Compared with other deposits of volcanic ashes along the “Cameroon Volcanic Line”, DAR and VN were selected as a result of their high amorphous phase (see Tables 3 and 4). After collection, they were washed in distilled water and then oven-dried at 105 °C for 24 h (Figure S0 in Supplementary Materials). They were ground and sieved below 75 µm to obtain DAR and VN powders consisting of low-density irregular particles.

**Figure 1:**
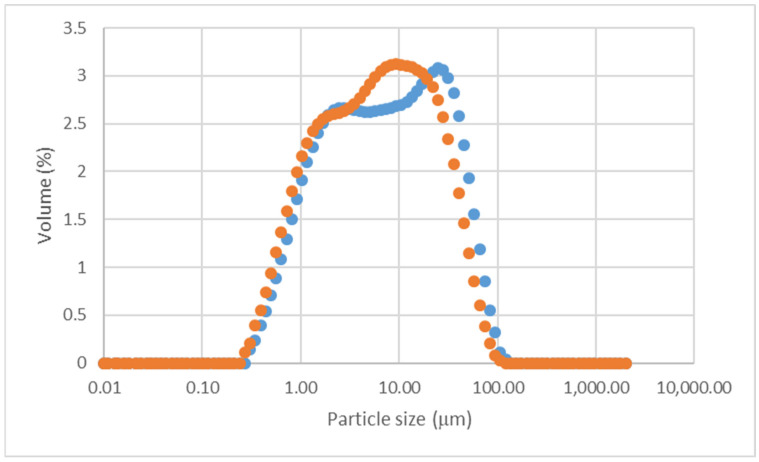
Comparison of particle size distribution curves of pure DAR (orange) and pure VN (blue) volcanic ash powders.

Granulometric analysis was performed by dynamic light scattering (DLS) using a water suspension measured in a Mastersizer 2000 granulometer (Malvern Instruments Ltd., Malvern, UK) (Figure 1).

From the comparison of the particle size distributions, it can be observed that both powders presented a 98% volume with particles in the 5–75 µm interval, indicating very limited aggregation. The two volcanic ash powders, DAR and VN, were then sieved to obtain the following two different particle size fractions: (i) DARm and VNm with a particle diameter Φ < 43 µm and (ii) DARM and VNM with a particle diameter 43 µm < Φ < 75 µm. The selection of two different particle sizes was fundamental in order to observe the possible different reactivities of the raw materials in a basic environment as a function of fineness. DAR and VN powders with particle sizes of 5–75 µm were also tested.

X-ray fluorescence spectroscopy (XRF; ARL Advant-XP automated X-ray spectrometer) was used to determine the chemical composition, in terms of major constituents, of the individual size fractions of pressed powder pellets of volcanic ash (Table 1). The matrix correction method proposed by Lachance and Trail [42] was applied. Volatile contents were determined as loss on ignition (L.O.I.) at 1100 °C, 2 h. The accuracy of the XRF data was evaluated using results from an international standard, which was run as unknown [43]. The results, including minor elements of the two volcanic ashes, are presented in Supplementary Table S1. The detection limits for the XRF analyses were evaluated using results from multiple runs of twenty-nine international standards [44].

The sodium silicate used had a mass composition of 27.09% SiO_2_ and 8.85% Na_2_O and pH = 11.7 (64.06% H_2_O; mass ratio SiO_2_/Na_2_O = 3.06 with a density of 1.373 g/cm^3^ at 20 °C) and was supplied by Ingessil S.r.I., Verona, Italy (Table 1). An approximate formula could be the following: 1Na_2_O·3SiO_2_·25H_2_O.

A sodium hydroxide solution of molarity = 12 M was obtained by dissolving NaOH flakes of a 96 wt% purity (supplied by Sigma-Aldrich Italia S.r.I., Milan, Italy) in distilled water. It was prepared and stored for 24 h before use. The activator solution was prepared 24 h before use and consisted of a mixture of a 2:1 volume ratio of Na-silicate to NaOH solution with a final SiO_2_/Na_2_O molar ratio of 1.24, similar to that optimised in the literature [45]. An approximate formula might be the following: 2Na_2_O·2.5SiO_2_·31H_2_O.

### 2.2. Leaching Test and Dissolution Kinetics

The dissolution kinetics of volcanic ash powders in a strong alkaline environment were investigated. Three NaOH solutions with different concentrations (8 M, 10 M, and 12 M) were prepared and placed in sealed polyethylene bottles 24 h before use. To better understand the dissolution kinetics, we varied the particle sizes of DAR and VN (Φ < 43 µm; 43 µm < Φ < 75 µm; and Φ < 75 µm). As temperature plays a key role in the dissolution of powders, the experiments were carried out at a temperature of 80 °C. Such a temperature was chosen as it was determined to be the best for the dissolution of oxides according to a former work by Djobo et al. (2016) [46].

The protocol consisted of measuring 2 g of powder in a beaker and dissolving it in 50 mL of NaOH at different times (20, 40, 60, 100, and 120 min). These different times were chosen because, in a previous study [46], the initial setting time for alkaline-activated ashes using the Vicat’s needle test took approximately 2 h. The beaker was then placed on a heated plate equipped with a magnetic stirrer. All dissolution tests were carried out using Polytetrafluoroethylene (PTFE) beakers to reduce the potential glass dissolution caused by NaOH. The sodium hydroxide solutions (8 and 10 M) were used to dissolve the as-received DAR and VN powders (Φ < 75 µm) at the following times: 20, 60, 100, and 120 min. The NaOH 12 M was used to dissolve the DAR and VN powders (Φ < 43 µm and Φ < 75 µm) at all times (20, 40, 60, 100, and 120 min). At the end of each time, the assembly was removed from the hot plate and filtered. The filtrate was collected, acidified (HNO_3_ 4% *v*/*v*) to a pH ranging from about 2 to 3, and then subjected to ICP-MS analysis to determine the contents of silicon, iron, aluminium, calcium, and magnesium dissolved during the dissolution process (standard solutions from Inorganic Ventures, Lakewood, NJ, USA, IV-ICPMS-71A). The solid residue was washed with distilled water to a neutral pH, dried, collected, and stored for further analysis (XRF, XRD, and FTIR).

### 2.3. Preparations of Geopolymer Specimens

The geopolymer samples, GP-DAR and GP-VN, were prepared from a mixture of sodium silicate/hydroxide (different concentrations) with the as-received (Φ < 75 µm) volcanic ash powders, DAR and VN, respectively. A mass ratio of activator solution/solid (wt/wt) of 0.41 for the DAR ash and 0.46 for the VN ash was used to ensure a very similar geopolymer formula, 1Na_2_O·1.5Al_2_O_3_·6SiO_2_·12H_2_O for GP-DAR and 1Na_2_O·1.3Al_2_O_3_·6SiO_2_·12H_2_O for GP-VN, and a very close Na_2_O/H_2_O ratio, i.e., to ensure very similar alkaline conditions. Since 8, 10, and 12 molar NaOH solutions were used, the geopolymer samples were labelled as GP-DAR 8, GP-DAR 10, and GP-DAR 12, respectively. The same is true for the geopolymer with VN ash, GP-VN 8, GP-VN 10, and GP-VN12. The masses of (DAR and VN) and the solutions used in this work are given in Table 2.

The mixture was homogenised using a mixer (Aucma 1400 W, Acuma Co., Ltd., Qingdao, China) for 5 min. The paste was poured into cubic moulds (25 × 25 × 25 mm^3^) and vibrated on a shaking table for 2 min to expel the air trapped by the fresh paste during mixing. The specimens in the moulds were carefully sealed in a thin polyethylene film and stored at the ambient laboratory temperature. Demoulding was carried out after full consolidation, i.e., after 7 days, as the L/S ratio was very high. The solidified geopolymers were cured at atmospheric temperature and humidity (25 °C, 50–60 RH%) [47]. To test the effect of curing temperature, some samples were solidified at 45 °C for 24 h before demoulding, being aware that a slight increase in curing temperature can induce an increase in mechanical properties [48]. Compressive strength and other tests were carried out on the consolidated geopolymers after 28 days of curing.

### 2.4. Characterisation of Hardened Geopolymers

#### 2.4.1. Reticulation Study by Water Resistance

To evaluate the overall chemical stability of the 3D aluminosilicate network reticulated in the final geopolymer products, the ionic conductivity of the eluate and the weight loss of the solid were measured according to a test procedure in water optimised in previous works [49,50]. MilliQ water (1:10 = solid/water weight ratio) was added to the ground geopolymer samples. After stirring, the solution was allowed to sediment for a short time before analysis. Ionic conductivity measurements were performed on the eluates using Crison GLP31 (Hach Lange Spain, S.L.U., Barcelona, Spain), at t1 = 0 h, t2 = 5 min, t3 = 10 min, t4 = 30 min, t5 = 2 h, t6 = 4 h, t7 = 6 h, t8 = 24 h, and t9 = 48 h, respectively.

#### 2.4.2. Reticulation by FT-IR Spectroscopy

Fourier transform infrared spectroscopy is one of the most sensitive characterisation techniques for the chemical bonding features of aluminosilicates. Several works have been carried out in this field on volcanic ash [45,46,48,51,52,53,54], so we also chose to complete the study of the degree reticulation with this technique.

FTIR spectroscopy was carried out on volcanic ash either in the form of powders (DAR and VN) or on powders of (GP-DAR12)- and (GP-VN12)-based geopolymers using a spectrophotometer (VERTEX 70, Bruker, Billerica, MA, USA) equipped with a deuterated sulphate detector with potassium bromide windows. The analysis was performed in the spectral range of 400–4000 cm^−1^ with a resolution of 4 cm^−1^ (scans).

#### 2.4.3. Mineralogical Investigation via XRD

The mineralogical phases of the volcanic ash and the resulting geopolymers were determined by powder X-ray diffraction using a Miniflex II (Rigaku, Matsubara-cho, Tokyo, Japan) with the following instrumental conditions: Cu Kα radiation; Ni filter; 2θ angle of 5–65°, angular step of 0.01° 2θ; step time of 5 s; divergence and antiscatter slits of 1°; and receiver slit of 0.2 mm. Phase identification was performed using the HighScorePlus software, version 3.0.5 based on the BGMN database (available at http://www.bgmn.de/index.html, accessed on 12 September 2024), while the quantitative analysis was obtained by the Rietveld method using the Profex software, version 5.3.1 [55], using zincite as an internal standard. The quality of the Rietveld refinements was assessed by both the visual observation of the observed vs. calculated patterns and by comparing the values of the discrepancy indices as the weight profile R-factor, R-weighted pattern—Rwp, whose values were less than 15% as an index of an adequate refinement [56].

#### 2.4.4. Microstructural Observation

Microstructural observations were carried out using an ESEM (ESEM-Quanta200-FEI, Hillsboro, OR, USA) equipped with EDS to assess the formation of the geopolymer amorphous phase and the presence of unreacted particles. The geopolymer samples to be analysed were cut, polished, and gold-plated before being placed in the analysis chamber to reduce charge accumulation and increase the penetration depth of the beam, thus improving the image quality. Once the sample was placed in the chamber, a secondary vacuum was applied, followed by electron beam scanning. The electron–matter interaction gave rise to various reactions (diffraction, scattering, and secondary electron emission). Images were obtained by collecting the secondary or backscattered electrons emitted from the material surface.

#### 2.4.5. Mechanical Properties

To test the mechanical properties of the DAR- and VN-based geopolymers, compression tests were carried out on 28-day-old specimens using a Controls L1052 testing machine (Cernusco, Italy). These measurements were made on cubic specimens. For each formulation, the compressive strength was the average of eight specimens. The tester was loaded to a limit of 30 kN. The specimen was subjected to continuous, progressive loading at an average rate of 1 mm/min. The tests were performed in the displacement control mode at a constant loading rate and without preload.

## 3. Results and Discussion

### 3.1. Dissolution Kinetics of the Volcanic Ash in Powder Form

#### 3.1.1. Chemical Composition of the Volcanic Ash Fractions

**Figure 2:**
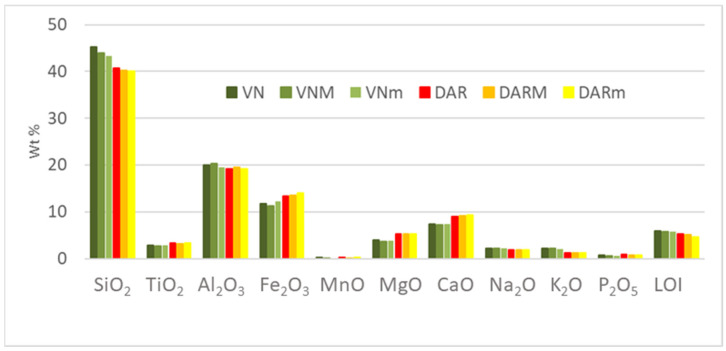
Chemical composition of the 3 different fractions of each volcanic ash: DAR (red colours) and VN (green colours). Numerical values are given in Table 1.

The three different particle size fractions of VN and DAR volcanic ash had very similar chemical compositions, as shown in Table 2 and Figure 2. The only possible observations were the slightly higher content of Fe in the fine fraction (DARm and VNm) and that the accuracy of the XRF data (Table 1, column indicated as error) was much lower than the natural variation of the ashes.

This observation of the homogeneity of the chemical composition distributed among the three different fractions indicates the absence of a mineral with excessive hardness combined with the absence of minerals with a high fragility.

#### 3.1.2. FT-IR of the Volcanic Ashes Before and After Alkaline Dissolution

Fourier transform infrared (FT-IR) spectroscopy has been used to analyse the environments of Al-O and Si-O bonds in aluminosilicates used as raw materials for alkali-activated materials or geopolymer synthesis. The as-ground particle size fractions, 5–75 µm, of both volcanic ashes, DAR and VN, were subjected to FT-IR observations before and after the NaOH dissolution. The highest NaOH concentration was chosen, 12 M, and the longer time, 2 h, to assure the most evident spectra variations between the materials before and after the alkali dissolution. 

**Figure 3:**
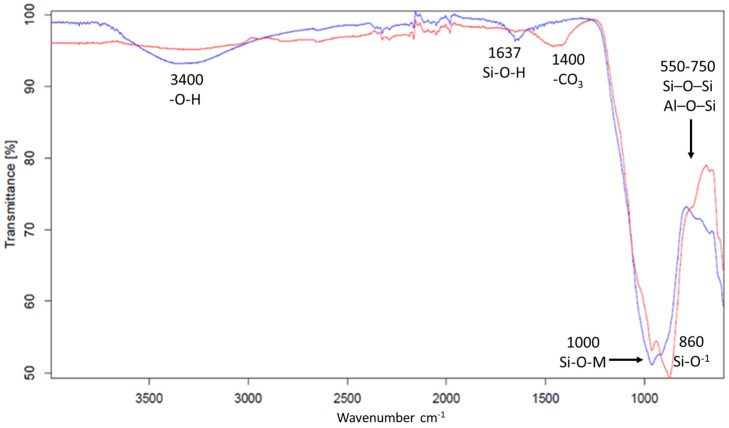
FT-IR spectra of DAR volcanic ash: as-ground (blue) and after immersion in NaOH 12 M for 2 h (red).

**Figure 4:**
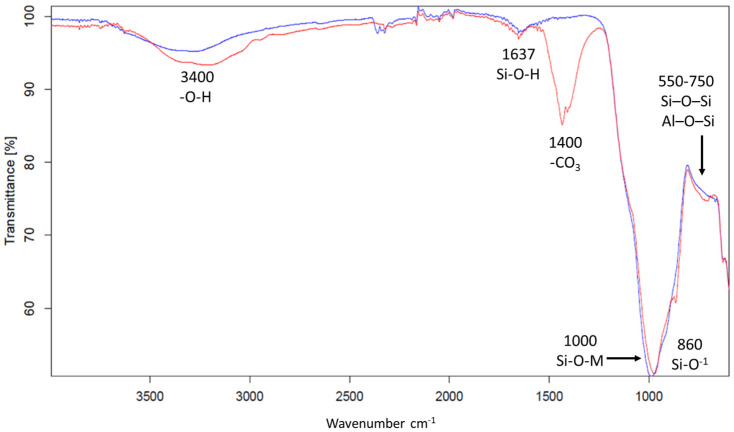
FT-IR spectra of VN volcanic ash: as-ground (blue) and after immersion in NaOH 12 M for 2 h (red).

The infrared spectroscopic results obtained from the as-ground volcanic ashes show very similar features (Figure 3 and Figure 4). The bands around 3200–3400 and 1637–1640 cm^−1^ correspond, respectively, to stretching vibrations of Si-O-H [52,57] and bending H-O-H bonds [46,58]. The main peak, located at 1000 cm^−1^, is attributed to the asymmetric stretching of the Si-O-M (with M = Si, Al) bond of the aluminosilicate framework, typical of the presence of amorphous aluminosilicate gel in binary systems [59,60,61]. The peak around 970 cm^−1^, which is more prominent in the DAR powder, and the peak around 1000 cm^−1^ can both be attributed to –Si–O–Si– bridging bonds in the geopolymer gel structure [62], whereas the peak around 860 cm^−1^ is attributed to Si–O terminal bond in the same structure [63]. The numerous peaks at around 600–500 cm^−1^ can indicate the presence of the ring vibrations of the Si–O bonds of silicate networks [46,58], and can be also ascribed to the symmetric stretching of Si–O–Si and Al–O–Si (550–750 cm^−1^) and bending Si–O–Si and O–Si–O (460–510 cm^−1^) vibrations [64,65], where octahedral aluminium, Al(VI), can be present [66].

The most obvious effect of dissolution is reported for the DAR volcanic ash, where the peak at about 1000 cm^−1^ is shifted to lower wavenumbers and the peak intensity for the vibrations at about 600–500 cm^−1^ decreases. The shift of the peak position of the main band characteristic of the Si-O-T (T:Si or Al) stretching vibrational bonds of the aluminosilicate framework to lower wavelength numbers is evidence of a decrease in the degree of polymerisation of the silicate system [67].

In addition, peaks around 1400 cm^−1^, indicative of carbonates, appear in the VA after the dissolution in NaOH. The Na^+^ ions that are not bound to the aluminosilicate network and remain in the pores solution can easily react with CO_2_ from the atmosphere to precipitate sodium carbonates [68].

#### 3.1.3. Reaction Kinetics in Alkaline Environment

The dissolution of DAR and VN volcanic ash in different aqueous NaOH concentrations was studied. Similar studies reported in the literature on more amorphous volcanic ashes with respect to DAR and VN report some percentages of dissolved silica over 2 or 2.5 h [69]. In the case of the VA studied, the amorphous fraction is lower, 50 for DAR and 74 wt% for VN (more data in Section 3.1.4), and the higher crystallinity makes dissolution more difficult.

**Figure 5:**
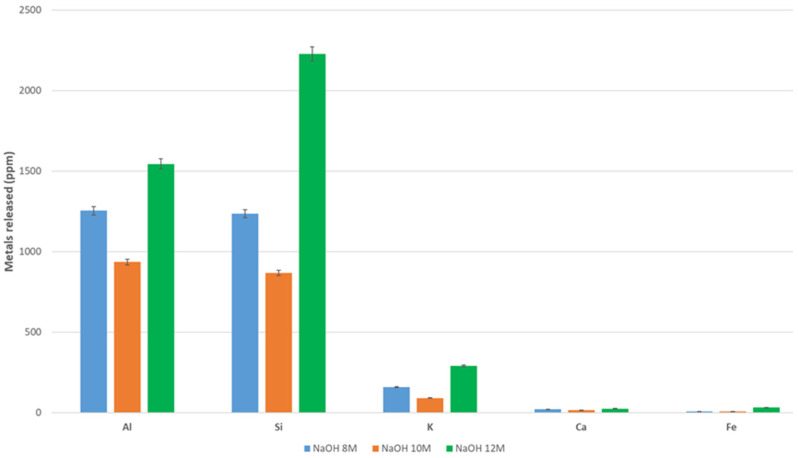
Leaching of metals of volcanic ash powder, DAR, after 8, 10, and 12 M after 120 min. The grain size used for the test is 5–75 µm.

As a general trend, we can observe that the values of the leachate elements of the most abundant oxides, namely Al, Si, K, Ca, and Fe, range from a few tens of ppm (Ca and Fe) to a few thousands of ppm (Al, Si, and K) (Figure 5), corresponding to a maximum concentration of 0.056 mol/litre (1520 ppm of Al) or 0.074 mol/litre (2100 ppm of Si). These values are far from those always higher than 1 mol/litre found in the literature for highly reactive aluminosilicates, such as rice hull ash, geothermal silica, fly ash, metakaolin, and granulated blast furnace slag [17].

Specifically, DAR leaches 1250, 950, and 1600 ppm for Al metal at 8, 10, and 12 M, respectively. These values are 1250, 900, and >2000 ppm for Si. VN leaches 1150, 600, and 950 ppm, respectively, at 8, 10, and 12 M for Al and 880, 400, and 1150 ppm for Si. It is observed that Al and Si dissolve rapidly in 12 M NaOH, presenting 750 ppm for both metals after 20 min of dissolution and 1500 and 2300 ppm for Al and Si, respectively, after 60 min, while 2000 and 3000 ppm are observed after 100 min. This trend shows a good dissolution potential of DAR samples with respect to VN, where the dissolution seems to be somewhat low, reaching only 1500 and 2000 ppm for Al and Si after 100 min. Si particles from DAR with a size of >75 µm are those with a high dissolution rate. A similar trend is observed for VN.

Please notice that an error was evaluated on two repeated tests and is related to the variability of the VA, as well as to the reproducibility of the test, not to the sensitivity of the ICP-MS equipment used.

**Figure 6:**
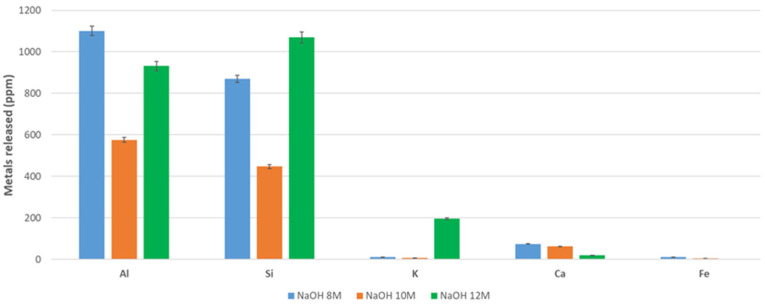
Leaching of metals of volcanic ash powder, VN, after 8, 10, and 12 M after 120 min. The grain size used for the test is 5–75 µm.

**Figure 7:**
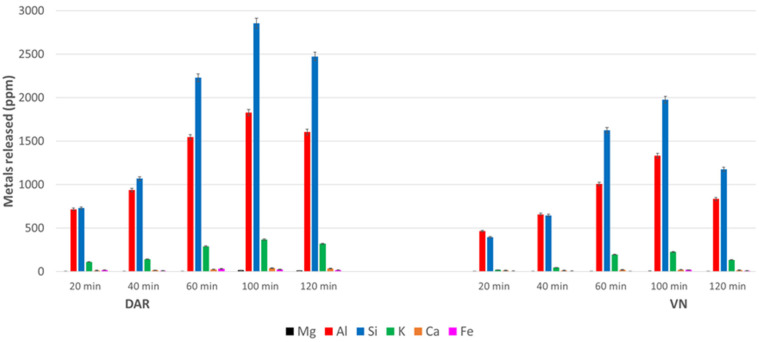
Leaching of metals of volcanic ash powders, DAR and VN, after immersion in NaOH 12 M as a function of time. The grain size used for the test is 5–75 µm.

Referring to previous work [46], the highest concentration of NaOH, 12 mol/L, seems to give the highest levels of elements released, although 8 M gives similar results. The alkali-activated materials could then be produced using NaOH 8 M with a similar efficacy to 12 M, but with limiting the cost and risk to operators. The amount of elements leached is not linearly proportional to the NaOH concentration (Figure 5 and Figure 6), whereas it is with the immersion time (Figure 7).

The results shown in Figure 7 are particularly interesting, as they clearly visualise the difference in leaching behaviour between the DAR and VN ashes. The DAR powder leaches a higher content of all elements, while VN leaches lower amounts, but both VAs reach the maximum around 100 min. From 100 min of immersion, the amount of leached element is almost constant or increases very slightly, probably at an equilibrium with the solid.

**Figure 8:**
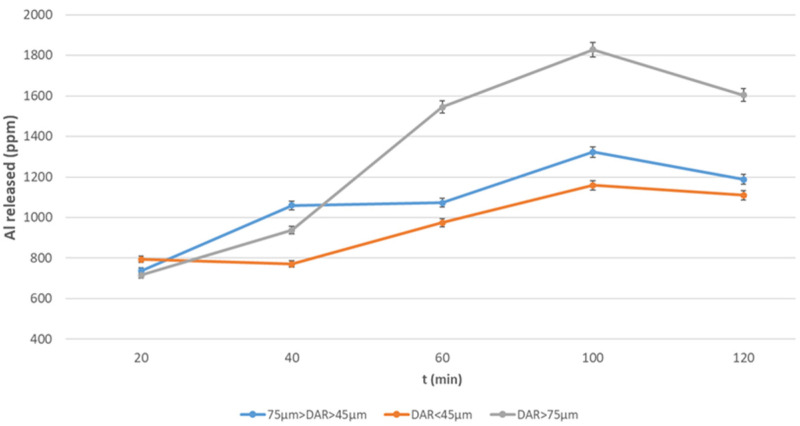
Al released from DAR volcanic ash at different grain size after NaOH 12 M over a period of 2 h.

**Figure 9:**
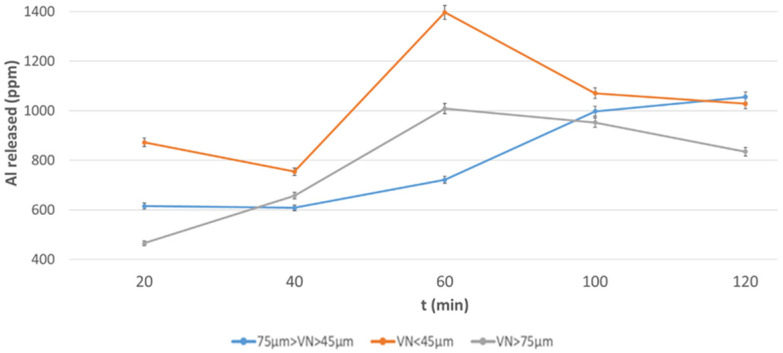
Al released from VN volcanic ash at different grain size after NaOH 12 M over a period of 2 h.

**Figure 10:**
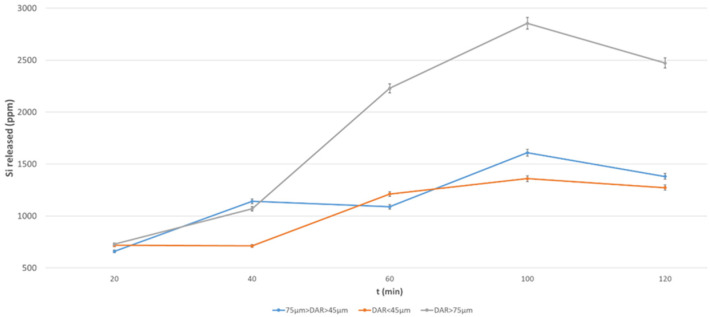
Si released from DAR volcanic ash at different grain size after NaOH 12 M over a period of 2 h.

**Figure 11:**
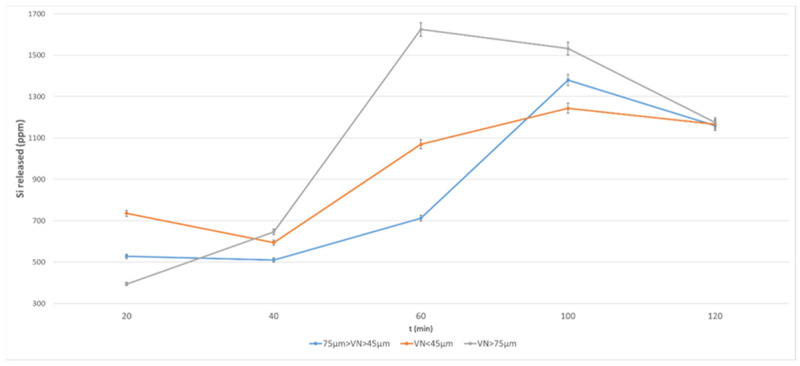
Si released from VN volcanic ash at different grain size after NaOH 12 M over a period of 2 h.

To better illustrate the dissolution process, the elements leached are also shown as a function of grain size (Figure 8, Figure 9, Figure 10 and Figure 11). Figure 8 and Figure 9 refer to the release of Al; this element is released in the highest amount from the DAR ash (higher concentration) with respect to the VN ash, but the finest fraction of VN ash (Φ < 43 µm) has the highest percentage of Al-bearing soluble components.

Figure 10 and Figure 11 show the data relating to Si release, where the same comments can be repeated, with the exception that none of the finer fractions have a reliable concentration of Si-bearing soluble phase.

From the element leaching data, it appears that the reactive fractions below 75 µm are all comparable, except for Al in VN. For the VN volcanic ash, it is important to retain the finest fraction when activating the powder with alkaline solution.

#### 3.1.4. Mineralogical Composition of the Volcanic Ash Fractions

The dissolution reaction at an extremely high pH and a low temperature (T = 80 °C) is a chemical process involving amorphous, semi-crystalline, and crystalline phases present in the VA, which were investigated with different analytical techniques, including X-ray powder diffraction [70]. This paragraph presents the fate of the mineralogical phases present in the two VA precursors before and after immersion in NaOH.

**Figure 12:**
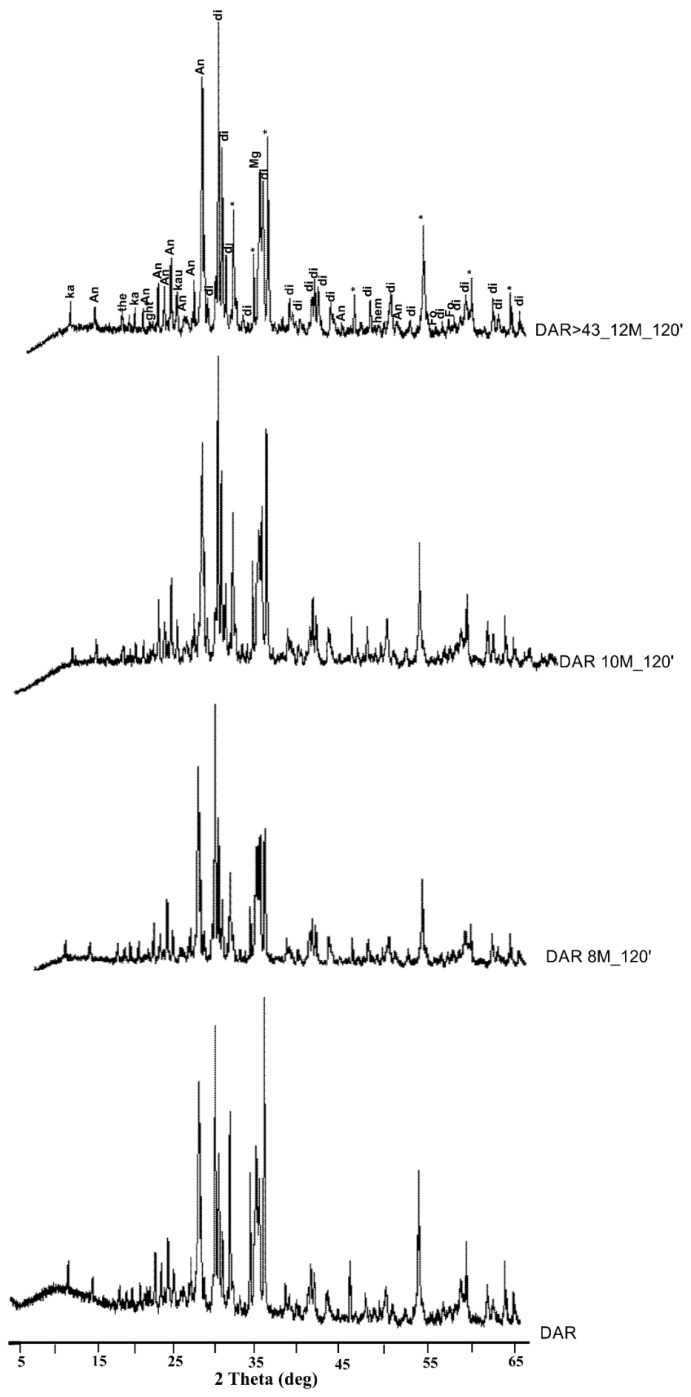
XRD patterns of DAR powders before and after immersion in NaOH at different molar ratios for 120 min. di = diopside (PDF: 19–0239); hem = hematite (PDF: 89–2810); An = Anorthite (PDF: 71–0748); Ka = Kaersutite (PDF: 44–1450); gibb = gibbsite (PDF: 96–101–1082); ght = goethite (PDF: 81–0462); Mg = Magnetite (PDF: 89–3854); Fo = Forsterite (PDF: 87–0619); Au = Augite (PDF: 71–1070); * = zincite (standard).

**Figure 13:**
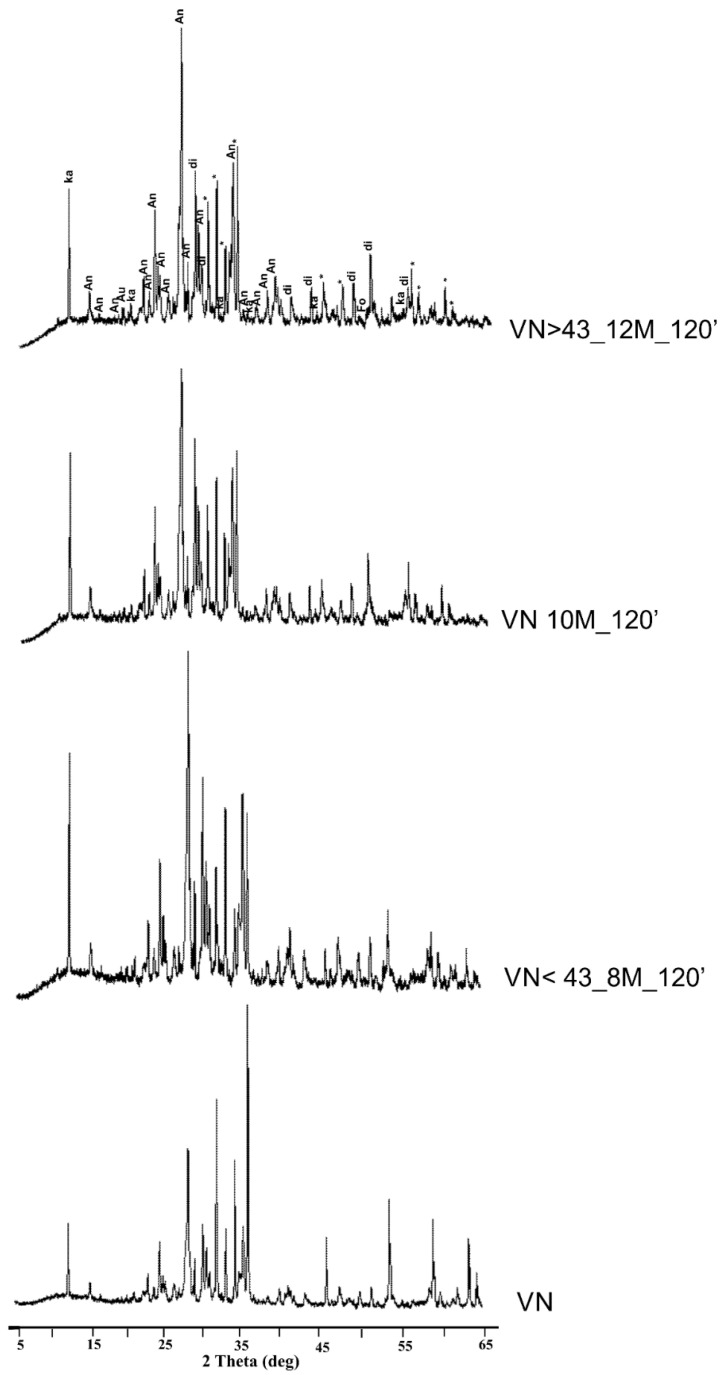
XRD patterns of VN powders before and after immersion in NaOH at different molar ratios for 120 min. di = diopside (PDF: 19–0239); hem = hematite (PDF: 89–2810); An = Anorthite (PDF: 71–0748); Ka = Kaersutite (PDF: 44–1450); gibb = gibbsite (PDF: 96–101–1082); ght = goethite (PDF: 81–0462); Mg = Magnetite (PDF: 89–3854); Fo = Forsterite (PDF: 87–0619); Au = Augite (PDF: 71–1070); * = zincite (standard).

Figure 12 and Figure 13 show the XRD patterns of the powders from the DAR and VN volcanic ash powders before and after 120 min of immersion in sodium hydroxide at different molar ratios (NaOH at 8, 10, and 12 M). At the same time, the XRD patterns of the volcanic ashes after alkaline dissolution with different molar ratios and at different time intervals can be observed in Figures S1–S4 in the Supplementary Materials. The mineralogical phases found in the DAR and VN raw materials are still present in the powders after NaOH treatment, indicating that these phases are inert to alkaline environments.

Specifically, the XRD patterns of DAR and VN show the presence of kaersutite, an amphibole with the following formula: NaCa_2_(Mg_3_AlTi^4+^)(Si_6_Al_2_)O_22_O_2_ [71], and the following five common crystalline phases: diopside CaMgSi_2_O_6_, anorthite sodian (Ca,Na)(Si,Al)_4_O_8_, augite (Ca,Mg,Fe)(Mg,Fe)Si_2_O_6_, magnetite Fe^2+^Fe^3+^_2_O_4_, and forsterite Mg_2_SiO_4_, which are typically found in volcanic ash and are consistent with the chemical analysis reported in Table 1 [72,73]. In addition, goethite is present in DAR. Traces of thénardite, Na_2_SO_4_, are present in some samples as a result of naturally occurring sulphur compounds in volcanic environments, whereas no new crystalline phases are detected in either the DAR or VN alkaline products after the reaction with NaOH at various molar ratios and time intervals.

In terms of the intensity peaks, all the crystalline phases initially present in DAR and VN were also found in the aluminosilicate products. However, in the DAR 8M_120′ and DAR 10M_120′ samples, the peak intensity of diopside and magnetite in the region of 32–37° in 2θ tended to decrease slightly in the alkaline products with an increasing molarity of NaOH with respect to the raw materials, indicating that these crystalline phases may have participated in the alkaline activation (Figure 12). With regard to VN, no decrease in intensity was observed with an increasing molarity of NaOH. The quantitative data confirm what was observed in the qualitative analysis. In fact, the phases identified in the raw materials are also present in the products immersed in alkaline NaOH solutions of different molarities, in which their contents remain almost unchanged, as shown in Table 3 and Table 4.

However, in the DAR 8M_120 and DAR 10M_120 samples, slight decreases in magnetite and diopside contents were observed, confirming the comments already made in Figure 12. The scarcity of soluble Fe cations leads us to think that Fe^+2/+3^-hydroxides’ precipitation occurred in this high-pH environment. It is conceivable that the elevated calcium (Ca) concentration documented by inductively coupled plasma mass spectrometry (ICP-MS) for the diopside-bearing sample (DAR) in comparison to the other sample (VN) can be attributed to the dissolution of diopside, despite its classification as a non-reactive crystalline phase [70]. A slight release, faster for Ca and slower for Mg, was observed in the pH range from 2 to 12, with a peak intensity decrease towards a high pH for pure diposide [74].

In general, the mineralogical phases identified are present in the same proportions in all the samples analysed, regardless of the variation in the molarity of the NaOH used, the immersion time, or the particle size of the powder used, as can be seen in Tables S2 and S3 of the Supplementary Materials.

### 3.2. Characterisation of Hardened Geopolymers

#### 3.2.1. Weight Loss and Ionic Conductivity of Hardened Samples

**Figure 14:**
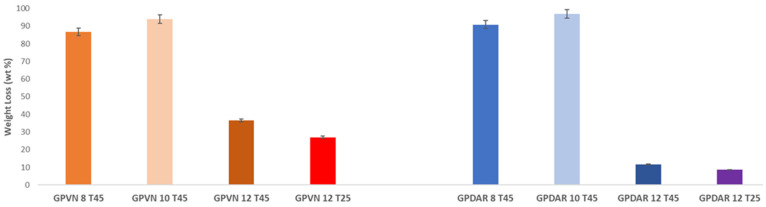
Weight loss of GP-DAR and GP-VN geopolymers cured for 24 h at two different temperatures (25 °C and 45 °C).

**Figure 17:**
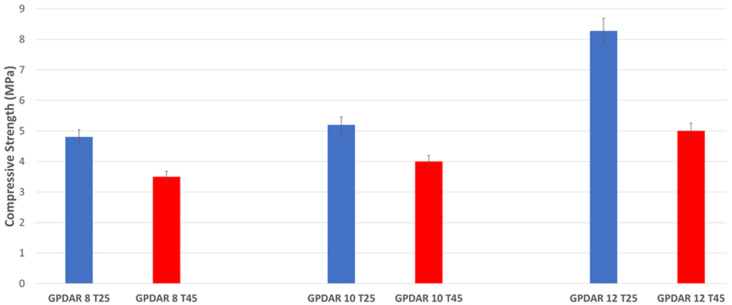
Comparison of mechanical properties of GPDAR samples made at different NaOH concentrations and different temperatures after 28 days of curing.

After performing weight loss tests (Figure 14), a temperature-related trend was observed in chemical resistance in the water of the cured specimens. Specifically, the geopolymers placed in an oven at 45 °C for 24 h showed a greater loss compared to the geopolymers cured at room temperature (25 °C), confirming some experimental results already reported in the literature [47]. Furthermore, it is evident that geopolymers containing both DAR and VN prepared with NaOH concentrations of 8 M and 10 M had a higher weight loss compared to the 12 M NaOH activation. This higher weight loss is indicative of a lower chemical stability, and, therefore, less efficient reticulation. This is also reflected in the mechanical properties (see also Figure 17).

**Figure 15:**
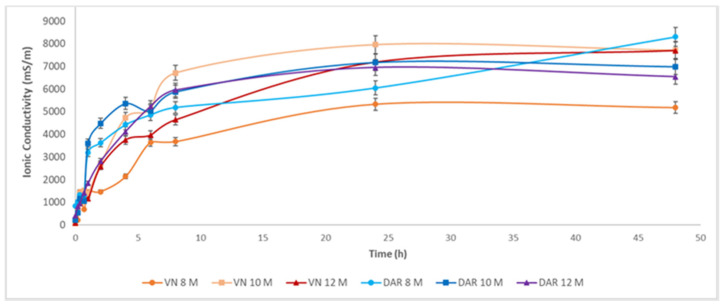
Ionic conductivity of geopolymer with DAR and VN made with different NaOH concentrations (8, 10, and 12 M), cured at 25 °C.

Figure 15 shows the ionic conductivity of the eluates obtained from the immersion in water of each individual geopolymer. The increase in the ionic conductivity of the volcanic ash geopolymer after 48 h can be attributed to the lower chemical stability of the geopolymer structure.

**Figure 16:**
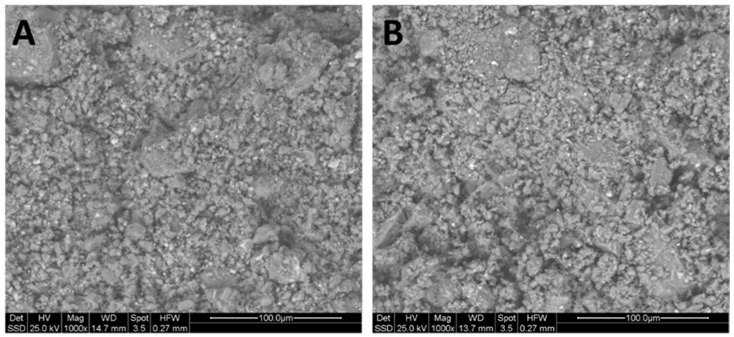
ESEM backscattered images of freshly fractured samples: (**A**) GPDAR 12 T25 and (**B**) GPVN 12 T25.

This increase in ionic conductivity over time in the solutions containing all the geopolymer samples can be attributed to several factors. As observed in the scanning electron microscope (SEM) images (Figure 16), complete geopolymerisation did not occur, probably influenced by the characteristics of the starting powders. It is noteworthy that, after 24 h, the values stabilised in almost all the leachates. It is interesting to observe that the samples prepared with DAR and VN using 8 M NaOH showed more significant deviations, while those prepared with 10 M and 12 M NaOH were closely clustered.

The range of values for the ionic conductivity of the eluates was significantly higher than that for similar geopolymers with volcanic ash and 10 to 25 wt% metakaolin [29], indicating that a small amount of metakaolin could increase the chemical stability also for this type of volcanic ash.

#### 3.2.2. Microstructure Observations

The dissolution of the amorphous/disordered fraction of VA in the alkaline liquor during geopolymer paste preparation led to the polycondensation of the powders into a dense block studied after 28 days of curing at room 25 °C, presenting the best performances (see Section 3.2.3). The microstructural changes were observed on the freshly fractured surfaces looking for the presence of a newly formed gel phase [75]. Figure 16A,B show the SEM analysis of GP-DAR T25 and GP-VN T25. Within the paste, the features include unreacted and partially reacted grains of the powders, forming a coexisting structure with a continuous mass of aluminosilicate. In particular, a significant proportion of the DAR and VN particles, particularly the larger ones, remain incompletely reacted, suggesting challenges in achieving full conversion during the geopolymerisation process. While the geopolymer matrix appears continuous and relatively dense, the SEM images reveal the presence of voids and cracks.

These structural irregularities, although subtle, have significant implications for the binding capacity and mechanical strength of the geopolymer, as can be seen in Figure 16B. Voids within the matrix indicate regions where geopolymerisation has not been completed, leaving unfilled spaces that could compromise the overall structural integrity of the material [76]. The presence of cracks in this geopolymer matrix raises concerns about the durability of the material and its ability to withstand external loads. These cracks can act as potential pathways for the ingress of harmful substances such as water or aggressive chemicals.

To densify the microstructure and improve its mechanical properties, more reactive components can be added to the volcanic ash, such as those indicated in the literature, like laterite [34] limestone [10,35], metakaolin [36,37], fly ash [77], kaolin, pozzolan and burnt lime [38] biomass-derived silica-rich ash [39], and finally, glass cullet [22,78].

#### 3.2.3. Mechanical Properties of Hardened Geopolymers

**Figure 18:**
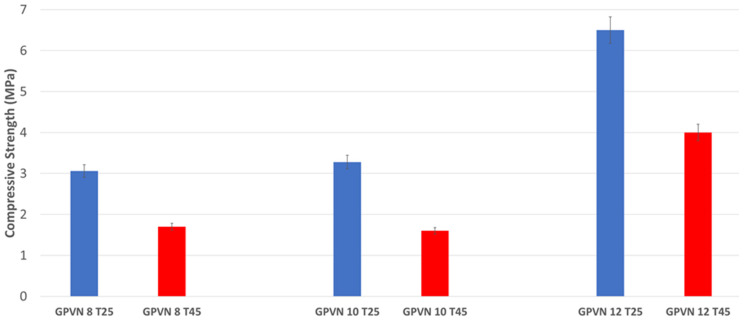
Comparison of mechanical properties of GPVN samples made at different NaOH concentrations and different temperatures after 28 days of curing.

Compressive strength tests were performed on all specimens after 28 days. Figure 17 and Figure 18 show all the compressive stress values of the prepared geopolymers. It can be observed that the samples prepared at 45 °C have a lower strength, regardless of the concentration of NaOH used, but in full agreement with the results of their chemical stability in water (see discussion of Figure 14). A general trend shows an increase in strength with the concentration of NaOH, corresponding to increased concentrations of monomeric Al and Si units in the solution capable of reticulating the aluminosilicate network. Furthermore, there is a significant difference between the DAR-based geopolymers and VN-based geopolymers, with the latter showing a worse mechanical performance. Presumably, the GP-DAR series has a high Si/Al ratio in the cured 3D aluminosilicate network, which, according to the literature, is associated with a higher degree of reticulation in stronger materials [79].

As indicated in a previous work focused on the curing temperatures of similar volcanic ashes consolidated via alkali activation, theses type of natural aluminosilicates require a thermal treatment during curing [48] to complete the geopolymerisation process at room temperature and present a mechanical resistance of around 30 MPa.

These alkali-activated VAs appear to be a good matrix for mortars and concretes for sustainable building materials, especially if local aggregates are added to increase the compressive strength at a low cost [7,80].

## 4. Conclusions

The dissolution kinetics in highly concentrated NaOH (8 to 12 M) were studied for two different volcanic ashes, which appeared to be of particular interest for the production of alkali-activated building blocks. The dissolution was studied at 80 °C for different times (10 to 120 min) in order to accelerate the leaching of soluble Si and Al species contained in the volcanic ash, compared to the slower dissolution that occurred in the same alkaline activator at room temperature during the preparation of the inorganic binder, also in the form of solidified blocks.

The high-temperature release allowed for the study of the eluates by ICP-MS and the characterisation of the undissolved volcanic ash by FT-IR and XRD. The results showed that the dissolution of the amorphous fraction (about 50 wt% for DAR and 70 wt% for VN) of the two volcanic ashes was preferred to the dissolution of the crystalline phases (mainly diopside and magnetite in DAR powders). The low amount of Fe detected in the eluate allowed us to hypothesise a precipitation of iron hydroxides insoluble in these extremely high-pH conditions. Also, in the FT-IR spectra, the DAR volcanic ash appeared more reactive, thus being more prone to produce soluble species that, with time, were more efficiently reticulated in the corresponding solid product.

The volcanic ashes dissolved at room temperature consolidated in 28 days into solid ceramic-like blocks with a fairly good chemical resistance, as tested by immersion in water for 24 h (average weight loss of about 10 wt%) and compressive strength tests (4–6 MPa). The microstructure was not very dense, suggesting the need for a more reactive additive in small quantities. Despite the difference in the dissolution rate, both volcanic ashes presented similar trend for geopolymerisation (similar values of compressive strength (5 MPa)). It was observed that increasing the concentration of NaOH improved the mechanical properties of the final geopolymer. In fact, more important was the dissolution, and significant was the potential of polycondensation and, consequently, the mechanical strength.

The three different grain sizes tested (Φ < 43 µm; 43 µm < Φ < 75 µm; and Φ < 75 µm) showed a very good homogeneity in the original chemical composition by XRF and the mineralogical composition by XRD, with a possible higher proportion of amorphous phase in the finer grains. Dissolution was more dependent on immersion time than on NaOH or grain size, suggesting a sustainable process in which the volcanic ash can be ground and sieved below 75 µm and activated with NaOH 8 M. The technology for producing building blocks from volcanic ash using mild alkaline dissolution with 8 molar NaOH followed by consolidation at room temperature was demonstrated.

According to the literature, the higher the content of reactive phase and better the ability of the aluminosilicate to dissolve in alkaline solution, the more reliable the properties of the products obtained, such as mechanical strength, durability, etc. The potential applications of geopolymers are many, as there are numerous types of building materials. The physical and mechanical properties of alkali-activated materials depend on the intrinsic properties of the aluminosilicate used and the activation process.

Considering that the materials tested in this study did not contain aggregates, their compressive strength could be reasonably increased. However, the limitation we can see for the application of VA-based materials in the field of construction is the lack of regulation. In addition, the limitations regarding the efficiency of these volcanic ashes to act as solid precursors for geopolymerisation include the extent of dissolution with a relatively high fraction of crystalline and metastable phases that would not participate in the chains of polysialates. Additionally, the high values of Si/Al make it present volcanic ashes as additives or raw solid precursors that need Al-rich additions to perform a good extent of geopolymer cement.

## Figures and Tables

**Table 1:** XRF data showing the major element composition (expressed as oxides in wt%) of all fractions of DAR and VN volcanic ashes. Minor elements are reported in Supplementary Table S1. Chemical data for sodium silicate and NaOH are those reported by the manufacturers.

wt%	VNm	VN	VNM	DARm	DAR	DARM	Error
SiO_2_	43.26	44.05	43.95	40.07	40.46	40.19	0.01
TiO_2_	2.93	2.85	2.69	3.35	3.26	3.22	0.08
Al_2_O_3_	19.60	19.51	20.34	19.25	19.12	19.60	0.07
Fe_2_O_3_	12.19	11.51	11.28	13.96	13.36	13.50	0.04
MnO	0.19	0.20	0.18	0.20	0.20	0.19	0.01
MgO	3.77	3.83	3.63	5.33	5.33	5.25	0.08
CaO	7.38	7.14	7.20	9.30	8.89	9.10	0.02
Na_2_O	2.16	2.19	2.20	1.87	1.83	1.87	0.09
K_2_O	2.12	2.20	2.17	1.17	1.19	1.19	0.04
P_2_O_5_	0.63	0.69	0.65	0.80	0.95	0.83	0.05
LOI (1100 °C, 2 h)	5.77	5.830	5.71	4.71	5.432	5.06	0.005
Total	100.0	100.0	100.0	100.0	100.0	100.0	
Sodium Silicate Solution							
SiO_2_			27.09		wt%		
Na_2_O			8.85		wt%		
H_2_O			64.06		wt%		
Total			100				
SiO_2_/Na_2_O			3.06		Weight ratio		
SiO_2_/Na_2_O			3.16		Molar ratio		
Na_2_O/H_2_O			0.14		Weight ratio		
Na_2_O/H_2_O			0.04		Molar ratio		
Concentration			35.94		wt%		
Density			1.373		g/cm^3^		
NaOH 12 M							
Na_2_O			27.05		wt%		
H_2_O			72.95		wt%		
Total			100				
Na_2_O/H_2_O			0.37		Weight ratio		
Concentration			34.91		wt%		
Density			1.375		g/cm^3^		

**Table 2:** Geopolymer formulations; L/S = liquid (NaOH solution + Na-silicate solution) to solid (volcanic ash) ratio by weight.

GP-DAR		
Volcanic ash DAR	70.82	wt%
NaOH	9.74	wt%
Na-silicate	19.45	wt%
Liquid/solid	0.41	Weight ratio
SiO_2_	33.91	wt%
Al_2_O_3_	13.53	wt%
Na_2_O	5.65	wt%
H_2_O	19.56	wt%
LOI + minor oxides	27.34	wt%
Total oxides + LOI	99.99	
SiO_2_/Al_2_O_3_	2.51	Weight ratio
Na_2_O/Al_2_O3	0.42	Weight ratio
Na_2_O/H_2_O	0.29	Weight ratio
Approx formula	1Na_2_O·1.5Al_2_O_3_·6SiO_2_ 12H_2_O	
**GP-VN**		
Volcanic ash VN	68.67	wt%
NaOH	10.45	wt%
Na-silicate	20.88	wt%
Liquid/solid	0.46	Weight ratio
SiO_2_	35.90	wt%
Al_2_O_3_	13.40	wt%
Na_2_O	6.18	wt%
H_2_O	21.00	wt%
LOI + minor oxides	23.52	wt%
Total oxides + LOI	100.00	
SiO_2_/Al_2_O_3_	2.68	Weight ratio
Na_2_O/Al_2_O_3_	0.46	Weight ratio
Na_2_O/H_2_O	0.29	Weight ratio
Approx formula	1Na_2_O·1.3Al_2_O_3_·6SiO_2_·12H_2_O	

**Table 3:** XRD mineralogical quantitative data of DAR volcanic ash powders. Kaer = kaersutite; Plag = plagioclase, Magn = magnetite; Diop = diopside; Then = thenardite; Forst = forsterite; Hem = hematite; Goet = goethite; Amor = amorphous.

ID Sample	Kaer	Plag	Magn	Diop	Then	Forst	Hem	Goet	Amor	TOT
DAR	2.70	13.84	3.60	22.22	0.60	4.76	-	0.52	51.76	100
DAR 8M_120	2.83	26.88	5.96	37.33	2.46	-	0.21	0.82	23.51	100
DAR 10M_120	2.55	22.02	3.55	29.96	0.80	4.96	0.3	0.20	35.66	100

**Table 4:** XRD mineralogical quantitative data of VN volcanic ash powders. Kaer = kaersutite; Plag = plagioclase, Aug = augite; Diop = diopside; Forst = forsterite; Amor = amorphous.

ID Sample	Kaer	Plag	Aug	Diop	Forst	Amor	TOT
VN	4.60	12.02	2.55	5.17	1.23	74.43	100
VN < 43_8M_120	10.14	22.05	3.19	12.00	1.40	51.22	100
VN 10M_120	16.11	38.52	14.34	14.06	2.73	14.24	100
VN > 43_120_12M	15.58	31.15	5.31	28.20	1.96	17.80	100

## Data Availability

The original contributions presented in the study are included in the article and Supplementary Materials, further inquiries can be directed to the corresponding author.

## Supplementary Materials

The following supporting information can be downloaded at: https://www.mdpi.com/article/10.3390/ma17205068/s1, Figure S0. Photos of DAR and VN volcanic ash, as received; Figure S1: XRD patterns of DAR > 43 powders before and after immersion in NaOH 12 M at different times; Figure S2: XRD patterns of DAR < 43 powders before and after immersion in NaOH 12 M at different times; Figure S3: XRD patterns of VN > 43 powders before and after immersion in NaOH 12 M at different times; Figure S4: XRD patterns of VN < 43 powders before and after immersion in NaOH 12 M at different times; Table S1: XRF data showing the minor element composition (expressed as ppm) of all fractions of DAR and VN volcanic ashes; Table S2: XRD mineralogical quantitative data of DAR volcanic ash powders; Table S3: XRD mineralogical quantitative data of VN volcanic ash powders.
