# Supplementary material for: Dissolution of Volcanic Ash in Alkaline Environment for Cold Consolidation of Inorganic Binders
Source: Materials (Basel). 2024 Oct 17;17(20):5068. doi: 10.3390/ma17205068 (PMC11509610; doi:10.3390/ma17205068)
Supplement: Supplementary file 1 [file materials-17-05068-s001.zip › materials-3231753-supplementary.pdf]

materials

MDPI

Supplementary Materials

Dissolution of Volcanic Ash in Alkaline Environment for Cold Consolidation of Inorganic Binders

Giovanni Dal Poggetto [1,2,†], Philippe Douwe [3,4,†], Antonio Stroscio [1,5,†], Elie Kamseu [1,6], Isabella Lancellotti [1], Antoine Elimbi [4] and Cristina Leonelli [1,*]

[1] Department of Engineering "Enzo Ferrari", University of Modena and Reggio Emilia, Via P. Vivarelli 10, 41125 Modena, Italy; giovanni.dalpoggetto@unimore.it (G.D.P.); antonio.stroscio@unimore.it (A.S.); elie.kamseu@unimore.it (E.K.); isabella.lancellotti@unimore.it (I.L.)
[2] Department of Civil, Construction, and Environmental Engineering, North Carolina State University, Raleigh, NC 27695-7908, USA
[3] Department of Mathematics-Physics-Chemistry, University of Pala, P.O. Box 20 Pala, Chad; philpsdouwe@yahoo.fr
[4] Department of Inorganic Chemistry, University of Yaoundé I, P.O. Box 334 Yaoundé, Cameroon; elimbiantoine@gmail.com
[5] Department of Biological, Geological and Environmental Sciences, University of Catania, Corso Italia 57, 95129 Catania, Italy
[6] MIPROMALO—Local Material Promotion Authority, P.O. Box 2396 Yaoundé, Cameroon
* Correspondence: cristina.leonelli@unimore.it
† These authors equally contributed to this manuscript.

SUPPLEMENTARY MATERIALS

Table S1. XRF data showing the minor element composition (expressed as ppm) of all fractions of DAR and VN volcanic ashes. Major elements are reported in Table 1. *Recommended values for international reference material BE-N are from Govindaraju (1994, Geostandard Newsletter, Special Issue, v. 118, 158 p.).

	VNm	VN	VNM	DARm	DAR	DARM	BE-N EXPER	BE-N *
**Ba**	1012	1005	1036	916	960	954	1102	1025
**Co**	31	31	35	38	40	39	58	60
**Cr**	110	130	104	185	207	164	376	360
**Cu**	30	39	28	40	55	42	95	72
**Ga**	30	31	30	29	31	31	17	17
**Hf**	9	9	9	11	9	8	5	5.6
**Nb**	96	102	97	107	108	110	102	105
**Ni**	64	61	61	145	134	135	266	267
**Pb**	3	4	4	6	4	3	3	4
**Rb**	42	45	43	34	36	35	52	47
**Sc**	13	23	11	10	30	10	27	22
**Sr**	978	1028	991	1171	1178	1180	1355	1370
**Th**	9	9	11	11	10	10	8	10.4
**V**	140	165	132	204	238	189	281	235
**Y**	31	27	27	37	26	27	26	30
**Zn**	139	145	127	139	135	132	150	120
**Zr**	489	415	428	540	392	416	257	260

Table S2. XRD mineralogical quantitative data of DAR volcanic ash powders. Kaer= kaersutite; Plag = plagioclase, Magn= magnetite; Diop= diopside; Then= thenardite; Forst= forsterite; Hem= hematite; Goet= goethite; Amor=amorphous.

ID sample	Kaer	Plag	Magn	Diop	Then	Forst	Hem	Goet	Amor	TOT
DAR	2.70	13.84	3.60	22.22	0.60	4.76	-	0.52	51.76	100
DAR <75 8M_120'	2.83	26.88	5.96	37.33	2.46	-	0.21	0.82	23.51	100
DAR <43	2.84	12.90	3.34	22.14	0.50	4.38	-	-	53.90	100
DAR<43_12M_20	1.68	9.68	2.04	13.23	0.60	1.66	-	0.40	70.71	100
DAR<43_12M_40	3.34	22.04	3.78	34.21	1.06	5.84	0.10	0.40	29.23	100
DAR <43_12M_60	5.34	19.96	5,00	30.13	2.06	5.41	0.40	0.70	31,00	100
DAR <43_12M_80	5.19	25.29	6.32	41.26	1.39	7.68	0.10	0.30	12.47	100
DAR<43_12M_100	2.26	9.64	2.06	14.01	0.60	2.42	-	0.30	68.71	100
DAR <43_12M_120	4.28	22.48	5.56	33.31	1.05	6.70	0.20	0.50	25.92	100
DAR <43 12M_100	2.96	22.17	5.71	37.73	1.33	6.21	0.20	0.20	23.49	100
DAR >43	2.08	19.67	4.34	26.89	0.74	4.03	0.20	0.50	41.55	100
DAR>43_12M_20	4.34	25.39	5.82	37.71	0.90	6.48	0.10	0.90	18.36	100
DAR>43 12M_60	4.62	25.63	5.92	35.05	0.90	6.55	0.16	0.30	20.87	100
DAR>43 12M_80	1.63	19.04	4.38	26.07	1.94	5.09	0.15	0.60	41.10	100
DAR>43_12M_100	2.90	22.17	5.99	33.39	0.80	6.78	0.40	0.40	27.17	100
DAR>43 12M_120	3.19	24.69	6.34	34.62	1.80	6.28	0.10	0.40	22.58	100
DAR <75 10M_120	2.55	22.02	3.55	29.96	0.80	4.96	0.30	0.20	35.66	100

Table S3. XRD mineralogical quantitative data of VN volcanic ash powders. Kaer= kaersutite; Anort= sodian-anorthite; Plag = plagioclase, Aug= augite; Diop= diopside; Forst= forsterite; Amor=amorphous.

ID sample	Kaer	Anort	Plag	Augite	Diop	Forst	Amor	TOT
VN	4.60	2.44	9.58	2.55	5.17	1.23	74.43	100
VN >43	14.24	10.53	20.42	2.16	20.53	1.70	30.42	100
VN > 43_ 20_12M	15.01	8.23	28.64	2.02	21.05	1.80	23.25	100
VN > 43_40_12M	15.03	7.05	35.50	-	21.13	2.08	19.21	100
VN > 43_ 60_12M	10.54	5.77	24.91	1.48	22.08	3.24	31.98	100
VN > 43_ 80_12M	10.71	6.32	24.10	5.30	19.02	2.41	32.14	100
VN > 43_100_12M	11.01	6.49	24.02	8.14	16.01	2.03	32.30	100
VN > 43_120_12M	15.58	11.81	19.34	5.31	28.20	1.96	17.80	100
VN <43	6.63	3.92	7.86	0.40	13.82	0.87	66.50	100
VN<43_20_12M	15.90	22.02	15.17	26.99	14.19	2.19	3.54	100
VN<43_40_12M	13.25	6.43	30.80	10.39	17.28	3.10	18.75	100
VN<43_80_12M	9.07	2.19	21.29	18.55	3.26	1.64	44.00	100
VN<43_100_12M	10.14	4.09	17.96	3.19	12,00	1.40	51.22	100
VN<43_120_12M	20.30	-	28.70	-	47.00	1.24	2.76	100
VN 10M_120	16.11	8.54	29.98	14.34	14.06	2.73	14.24	100

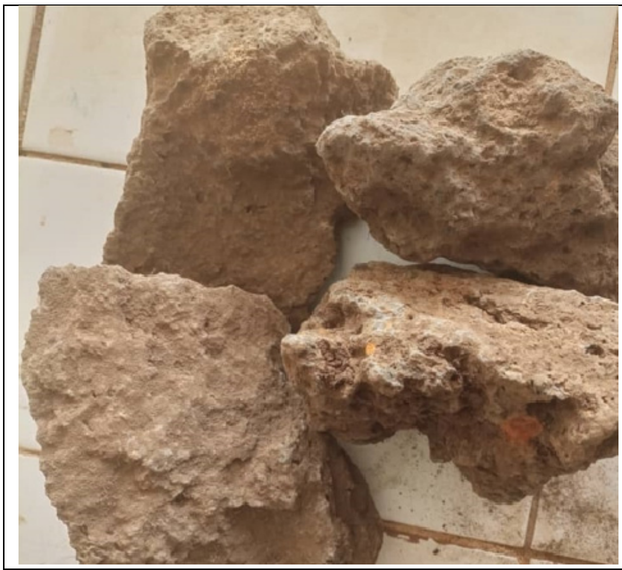	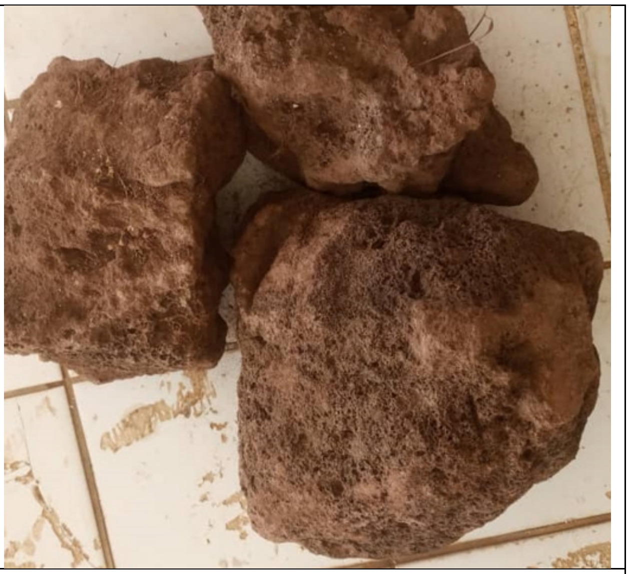
DAR	VN

Figure S0. XRD patterns of DAR >43 powders before and after immersion in NaOH 12M at different times. di= diopside; hem= hematite; An= Anorthite; Ka= Kaersutite; gibb= gibbsite; ght= goethite; Mg= Magnetite; Fo= Forsterite; Au= Augite; *= zincite (standard);.

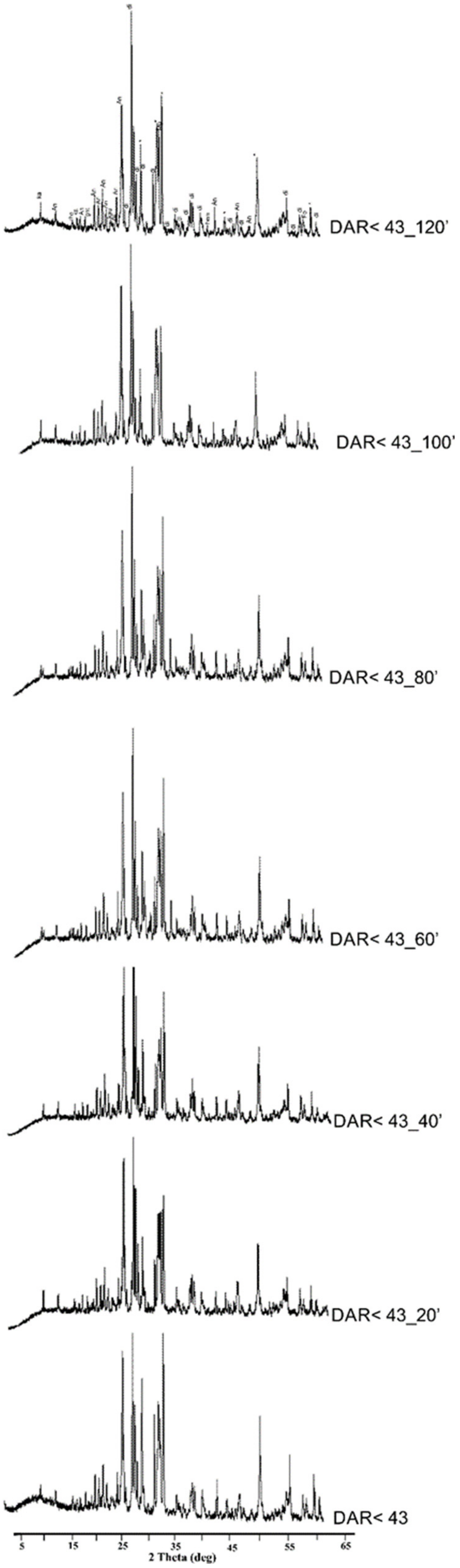

Figure S2. XRD patterns of DAR <43 powders before and after immersion in NaOH 12M at different times. di= diopside; hem= hematite; An= Anorthite; Ka= Kaersutite; gibb= gibbsite; ght= goethite; Mg= Magnetite; Fo= Forsterite; Au= Augite; *= zincite (standard).

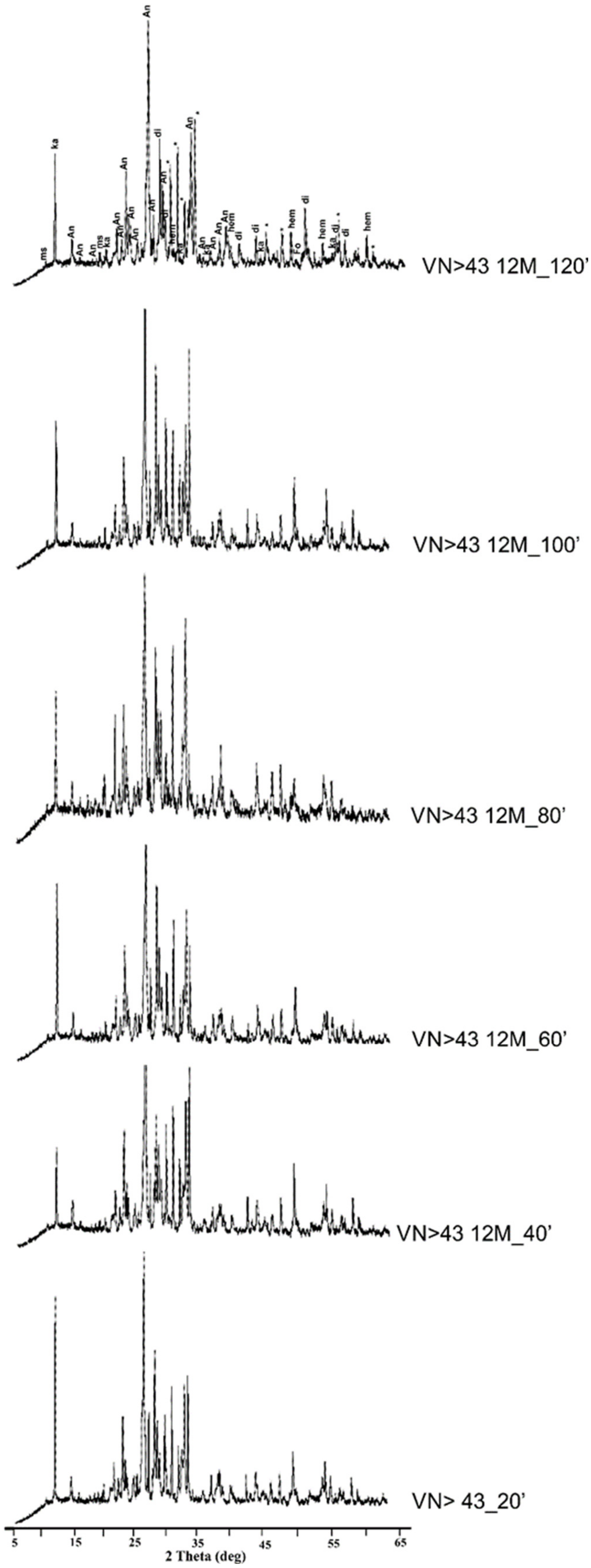

Figure S3. XRD patterns of VN >43 powders before and after immersion in NaOH 12M at different times. di= diopside; hem= hematite; An= Anorthite; Ka= Kaersutite; gibb= gibbsite; ght= goethite; Mg= Magnetite; Fo= Forsterite; Au= Augite; *= zincite (standard).

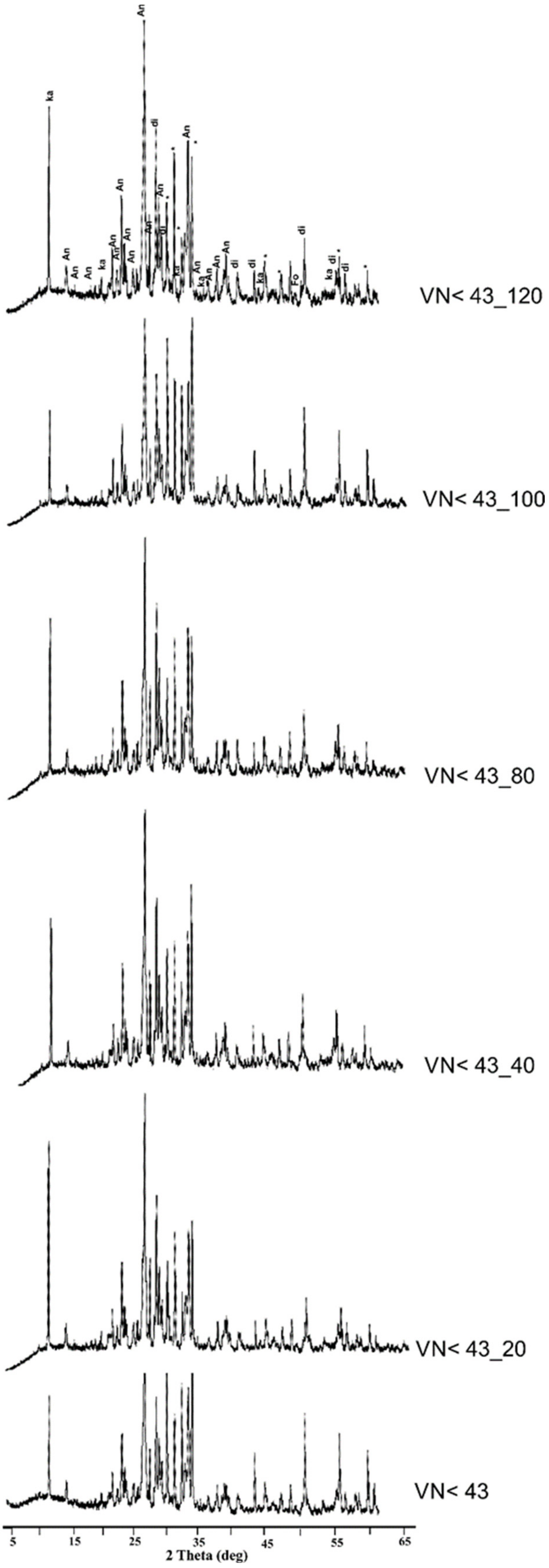

Figure S4. XRD patterns of VN <43 powders before and after immersion in NaOH 12M at different times. di= diopside; hem= hematite; An= Anorthite; Ka= Kaersutite; gibb= gibbsite; ght= goethite; Mg= Magnetite; Fo= Forsterite; Au= Augite; *= zincite (standard).
